# Treatment for complex knee osteoarthritis with total knee arthroplasty via lateral parapatellar approach-four case reports

**DOI:** 10.1097/MD.0000000000050598

**Published:** 2026-09-11

**Authors:** Shuailei Li, Boning Yang

**Affiliations:** a Department of Artificial Joint Revision Center, Luoyang Orthopedic-Traumatological Hospital of Henan Province (Henan Provincial Orthopedic Hospital), Zhengzhou, Henan, China.

**Keywords:** lateral parapatellar approach, medial parapatellar approach, total knee arthroplasty

## Abstract

**Rationale:**

It was previously believed that lateral parapatellar approach was only considered in the presence of valgus deformity, with much technical difficulties and complications. However, this approach may serve as an optional alternative for carefully selected complex knee cases when performing total knee arthroplasty (TKA) under certain clinical conditions

**Patient concerns:**

All 4 patients suffered from knee joints pain and limited mobility range from 1 to 10 years. There was no history of unusual diseases, infectious diseases or hereditary disorders.

**Diagnoses:**

Primary or secondary knee osteoarthritis.

**Interventions:**

All 4 patients underwent TKA under general anesthesia. The lateral approach was used in 3 cases, and the remaining case chosen the medial approach.

**Outcomes:**

Patients successfully received TKA with satisfactory restoration of lower limb alignment postoperatively. Three cases achieved good outcomes, demonstrating significant improvement in knee range of motion without complications. One case treated with the medial approach developed poor wound healing with subsequent infection.

**Lessons:**

The lateral parapatellar approach can be regarded as a feasible surgical choice for specific complex knee scenarios during TKA.

## 1. Introduction

Total knee arthroplasty (TKA) is a standard surgical treatment for end-stage knee osteoarthritis, effectively restoring function and alleviating pain.^[1,2]^ With an aging population, its application continues to grow. Since its inception, TKA techniques and surgical approaches have evolved significantly.^[3]^

In most cases, the medial parapatellar approach (MPA) is still the preferred choice among orthopedic surgeons.^[4]^ However, in knees with moderate-to-severe valgus deformity, its use presents specific challenges. Chronic valgus malalignment leads to lateral soft tissue contracture and medial collateral ligament attenuation. Employing the MPA in this setting can exacerbate medial laxity, often necessitating extensive lateral release, thicker polyethylene inserts, or constrained implants to achieve balance, which may compromise long-term outcomes.^[5]^ Furthermore, associated patellar maltracking often requires lateral retinacular release, risking patellar devascularization and anterior knee pain.^[6]^

To address these limitations, the lateral parapatellar approach (LPA) was introduced for valgus knees.^[7]^ Subsequent studies have demonstrated its advantages over the MPA in correcting deformity, reducing operative time, and expanding implant options.^[8,9]^ Despite this, concerns regarding technical difficulty, patellar eversion, and wound healing have limited its widespread adoption.^[10]^

This case series aims to illustrate specific clinical scenarios where the LPA may offer advantages over the medial approach and warrants further research and clinical application.

## 2. Case series

### 2.1. Case 1

A 62-year-old female patient was sent to the Henan Provincial Orthopedic Hospital with a history of severe right knee pain that had persisted for the past 10 years. The patient had intermittently taken oral non-steroidal anti-inflammatory drugs (NSAIDs) over the past decade, with progressive worsening of pain and valgus deformity. She presented to our hospital for further evaluation and management.

The patient has a 6-year history of hypertension managed with oral calcium channel blockers, currently demonstrating well-controlled blood pressure. She has undergone 2 prior surgical interventions for knee trauma. There is no history of blood transfusions or allergies to food or medications. The patient denies smoking, alcohol consumption, or exposure to toxic substances, dust, or radioactive materials. Additionally, she reports no family history of genetic disorders or infectious diseases.

**Figure 1.**
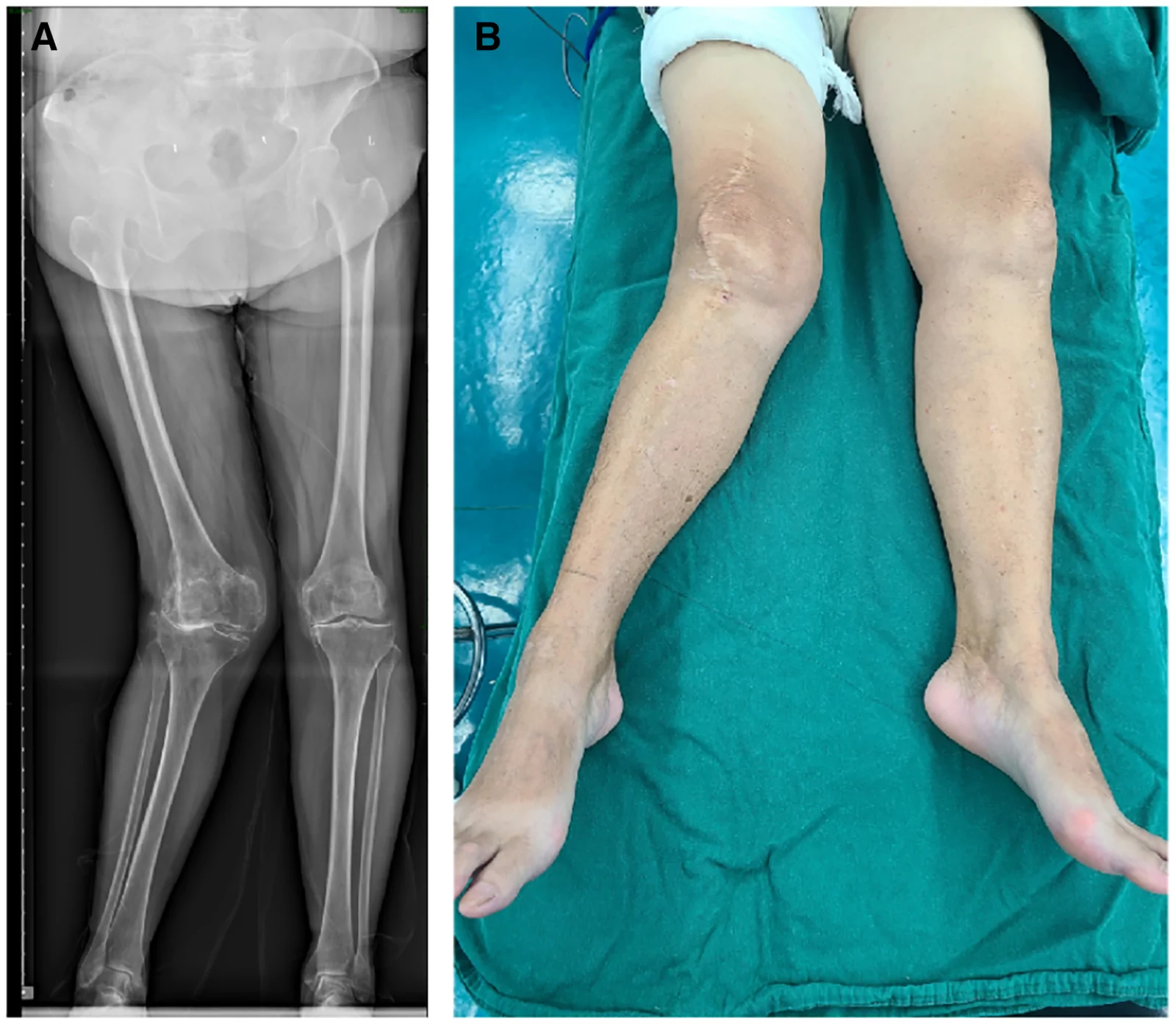
(A) Preoperative full-length radiograph of both lower extremities shows lateral femoral condyle defect and valgus deformity. (B) Preoperative physical examination revealed a surgical scar on the right lateral knee.

Physical examination revealed a right knee flexion valgus deformity with a well-healed 20 cm longitudinal surgical scar over the lateral parapatellar region, without signs of erythema or warmth. Tenderness was elicited over the lateral joint line; anterior and posterior drawer tests were negative, and the patellar grind test was positive. Right knee range of motion: 15 to 95 degrees. The Hospital for Special Surgery (HSS) knee score on presentation was 53 points. Radiographic examination suggested a defect in the lateral femoral condyle and valgus deformity of the right knee (Fig. 1). Other parameters, such as routine analysis of blood and urine, were normal.

**Figure 2.**
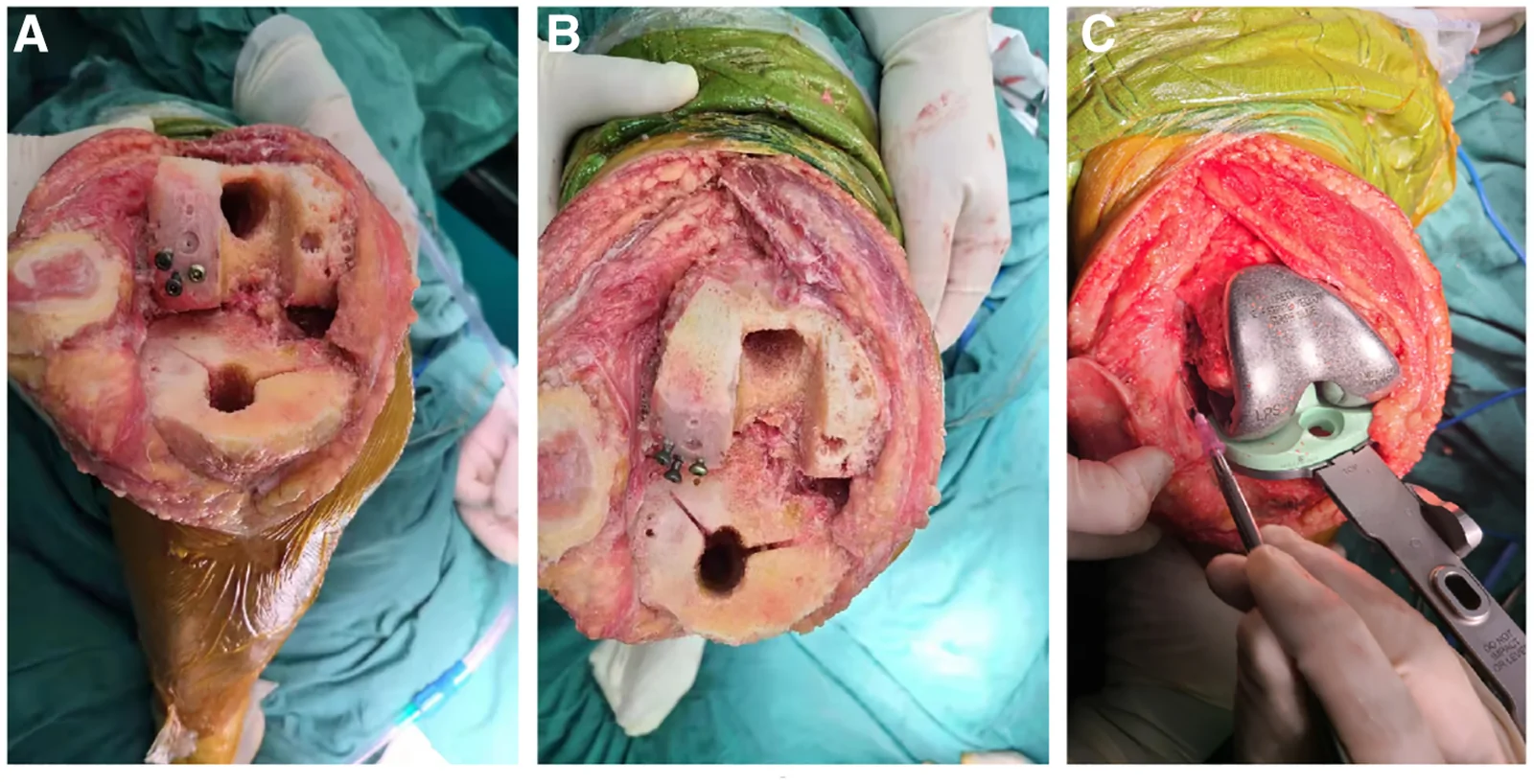
Intraoperative photograph with implants in situ. (A) and (B) addressing bone defects through screw implantation in the femoral condyle. (C) Pie-crusting technique for lateral soft tissue release.

The diagnoses of the case were type-Ⅲ valgus deformity, post-traumatic osteoarthritis, and hypertension. TKA was performed under general anesthesia, with strict aseptic protocols implemented preoperatively following standardized steps. Intraoperatively, the MPA was utilized based on the surgical team’s familiarity, and the original scar was not incorporated. Following adequate exposure, osteotomy was performed using the gap-balancing technique. The lateral femoral condyle defect was addressed via screw-reinforced bone grafting. The pie-crusting technique was applied to release the contracted lateral collateral ligament and iliotibial band (Fig. 2). Given the severe valgus deformity and compromised soft tissue integrity, a constrained condylar knee prosthesis was selected. Post-cement polymerization, the operated knee demonstrated satisfactory range of motion and stability.

**Figure 3.**
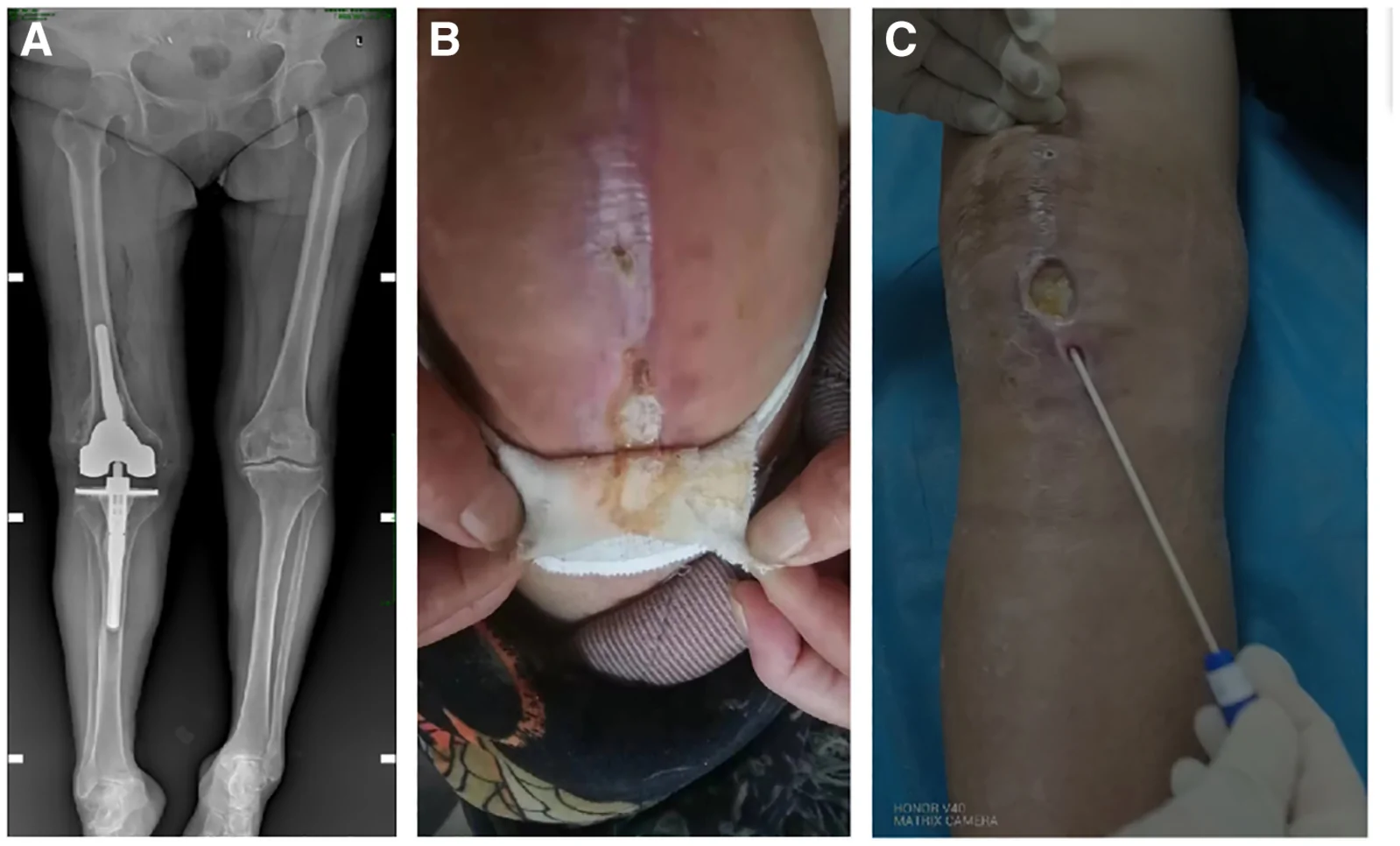
(A) Postoperative radiograph of full-length lower limb. (B) and (C) postoperative photos show poor wound healing at the incision site, ultimately leading to ulceration.

Postoperative radiographic evaluation demonstrated optimal alignment and positioning of the prosthetic components, with satisfactory restoration of the lower limb mechanical axis. However, the patient developed poor wound healing postoperatively, subsequently progressing to cutaneous ulceration (Fig. 3). Bacterial culture of wound exudate identified *Staphylococcus aureus* infection. This raises the question of whether the observed complication might correlate with inappropriate incision selection during the index procedure.

### 2.2. Case 2

A 53-year-old male patient was admitted to our department with right knee joint pain and limited mobility for 5 years, which had worsened over the past year. The patient had a history of intra-articular injection therapy and had intermittently taken oral NSAIDs. Due to poor recent outcomes, he came to our hospital for evaluation and sought surgical treatment.

The patient has a 5-year history of metabolic bone disease and has been regularly taking vitamin D and calcium supplements. There is no history of blood transfusions or allergies to food or medications. Besides, he has a 40-year history of smoking and alcohol consumption, but he reports no family history of genetic disorders or infectious diseases.

**Figure 4.**
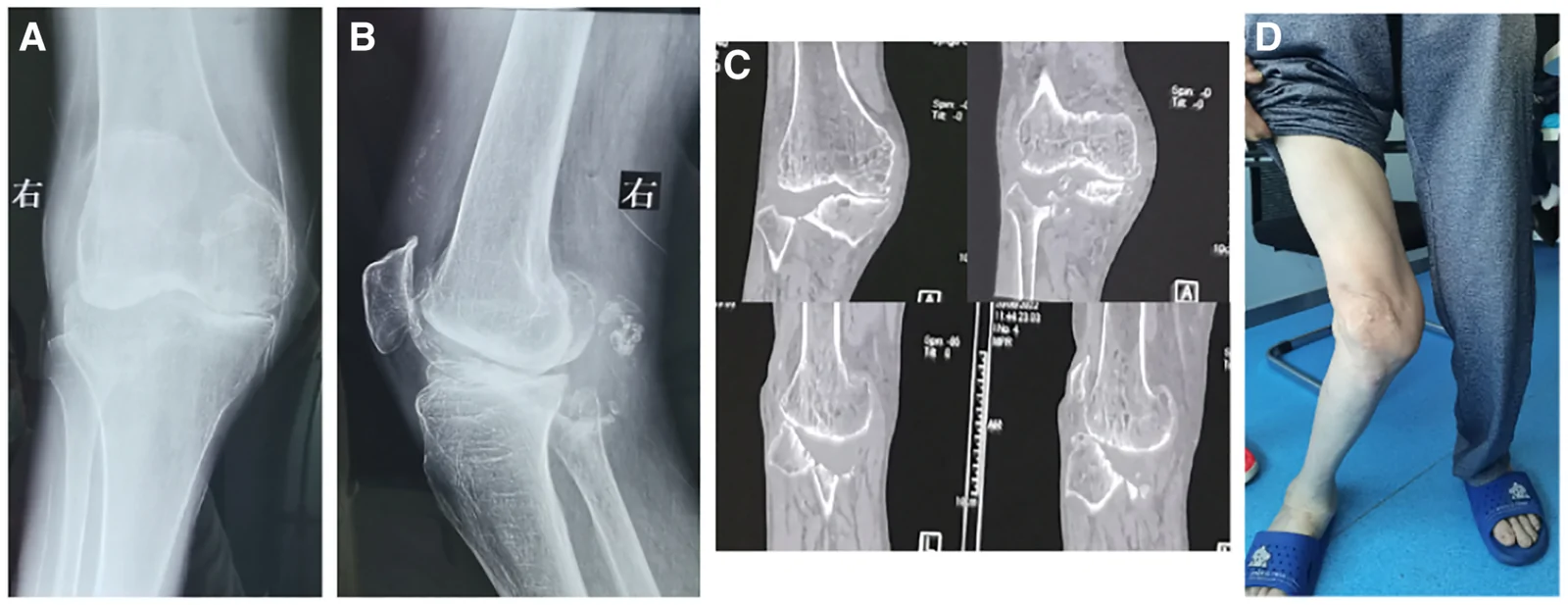
(A) Preoperative anteroposterior view radiograph of the right knee. (B) Preoperative lateral view radiograph of the right knee. (C) Non-contrast CT scan of the right knee joint reveals a bone defect in the lateral tibial plateau. (D) Preoperative anterior view of the patient’s right knee.

Physical examination demonstrated an irreducible valgus deformity of the right knee joint. Tenderness was elicited over the lateral joint line; anterior and posterior drawer tests were negative, and the patellar grind test was positive. Right knee range of motion: 5 to 100 degrees. The HSS score was 61 points. Radiographic examination reveals valgus deformity of the right knee joint combined with lateral tibial plateau bone defect (Fig. 4).

**Figure 5.**
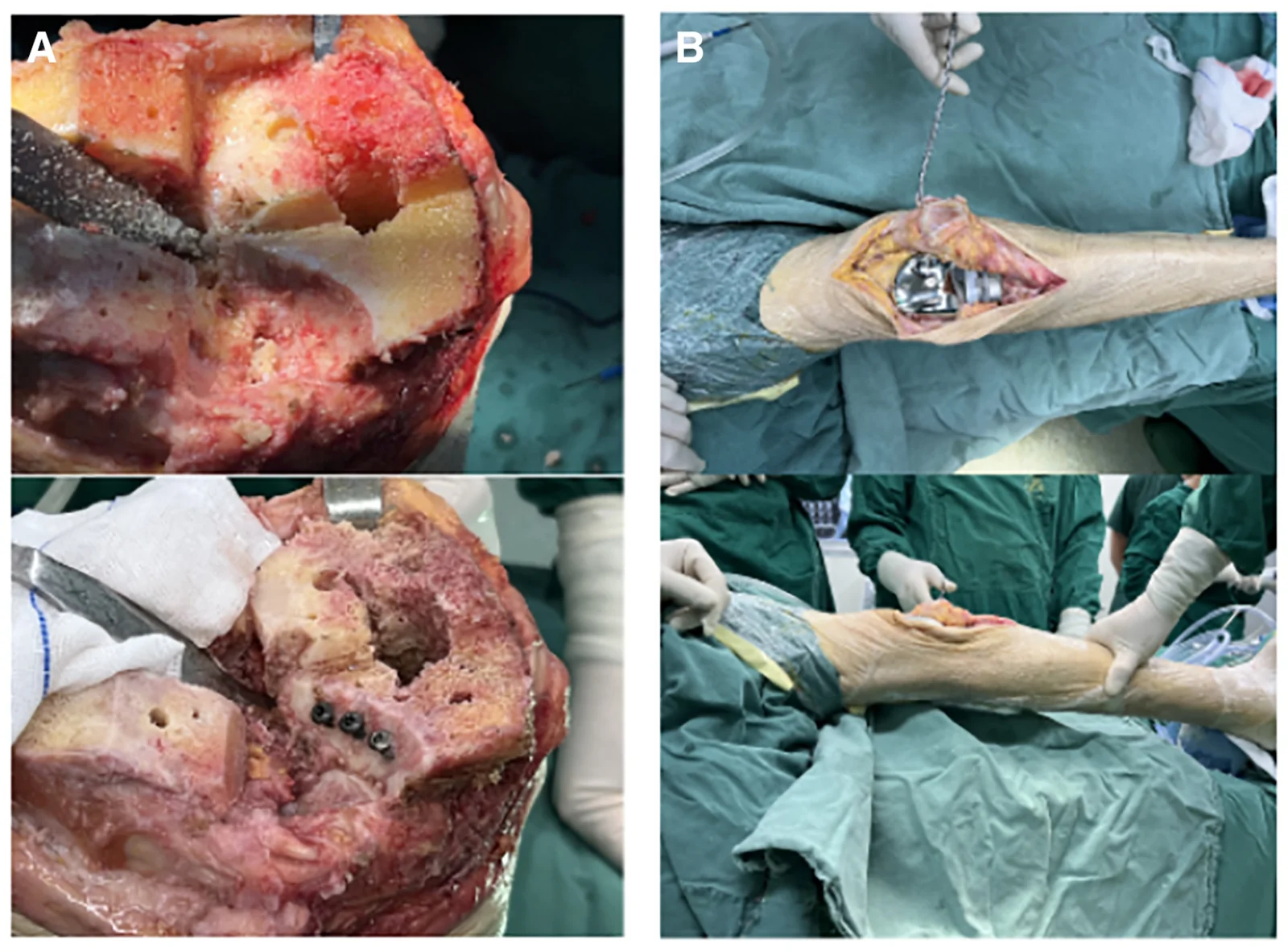
(A) Intraoperative photograph shows the restoration of the bone defect via screw implantation. (B) Intraoperative photos demonstrate successful restoration of the lower limb alignment.

The diagnoses of this case were type-Ⅲ valgus deformity and metabolic bone disease. We administered a second-generation cephalosporin intravenously 30 minutes preoperatively for infection prophylaxis. The TKA was performed under general anesthesia through a slightly lateral midline skin incision over the knee. A “Z”-shaped capsulotomy was made 1 cm lateral to the patella, releasing the capsule and lateral retinaculum. The distal femoral osteotomy was performed with 5° of valgus alignment. Following completion of the tibial osteotomy, a bone defect persisted in the lateral plateau. This was addressed using the screw-reinforced bone cement technology (Fig. 5). Although the case was diagnosed as type-Ⅲ valgus deformity, adequate gap-balancing was achieved through a lateral approach involving extensive release of the iliotibial band, posterolateral joint capsule, and lateral collateral ligament. Consequently, a posterior-stabilized prosthesis was selected for this patient, with only a tibial extension stem added as supplementary augmentation.

**Figure 6.**
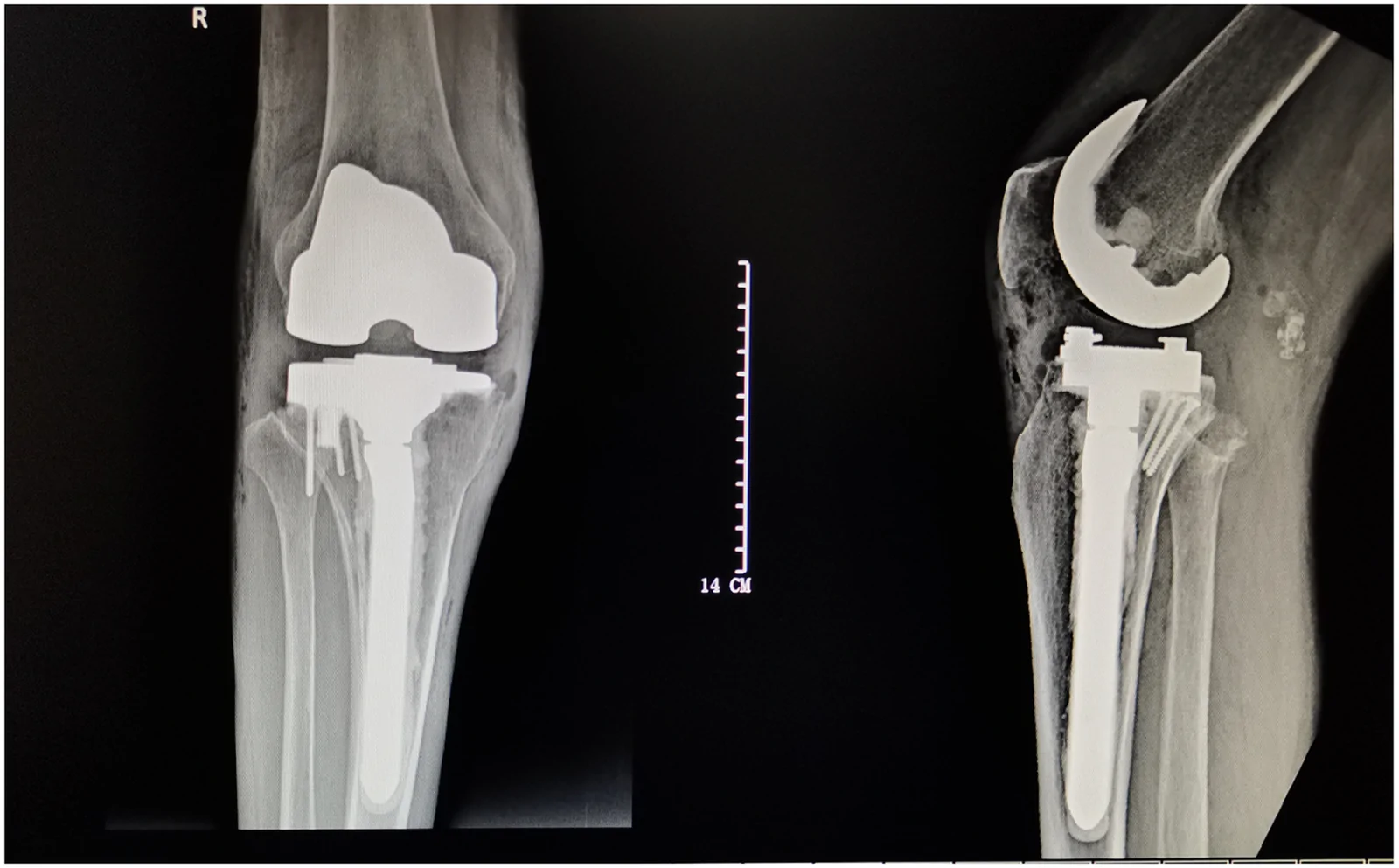
Postoperative anteroposterior and lateral view radiograph of the right knee.

Postoperative radiographic evaluation demonstrated optimal alignment and positioning of the prosthetic components (Fig. 6). At the 1-year postoperative follow-up, the latest HSS score was 90 points, and the patient was satisfied with the surgical outcome.

### 2.3. Case 3

A 63-year-old male patient complained of right knee pain with limited mobility for 1 year. The patient sustained a right tibial plateau fracture due to a traffic accident 1 year ago and regularly took oral NSAIDs intermittently with unsatisfactory results. He presented to our hospital for further treatment.

**Figure 7.**
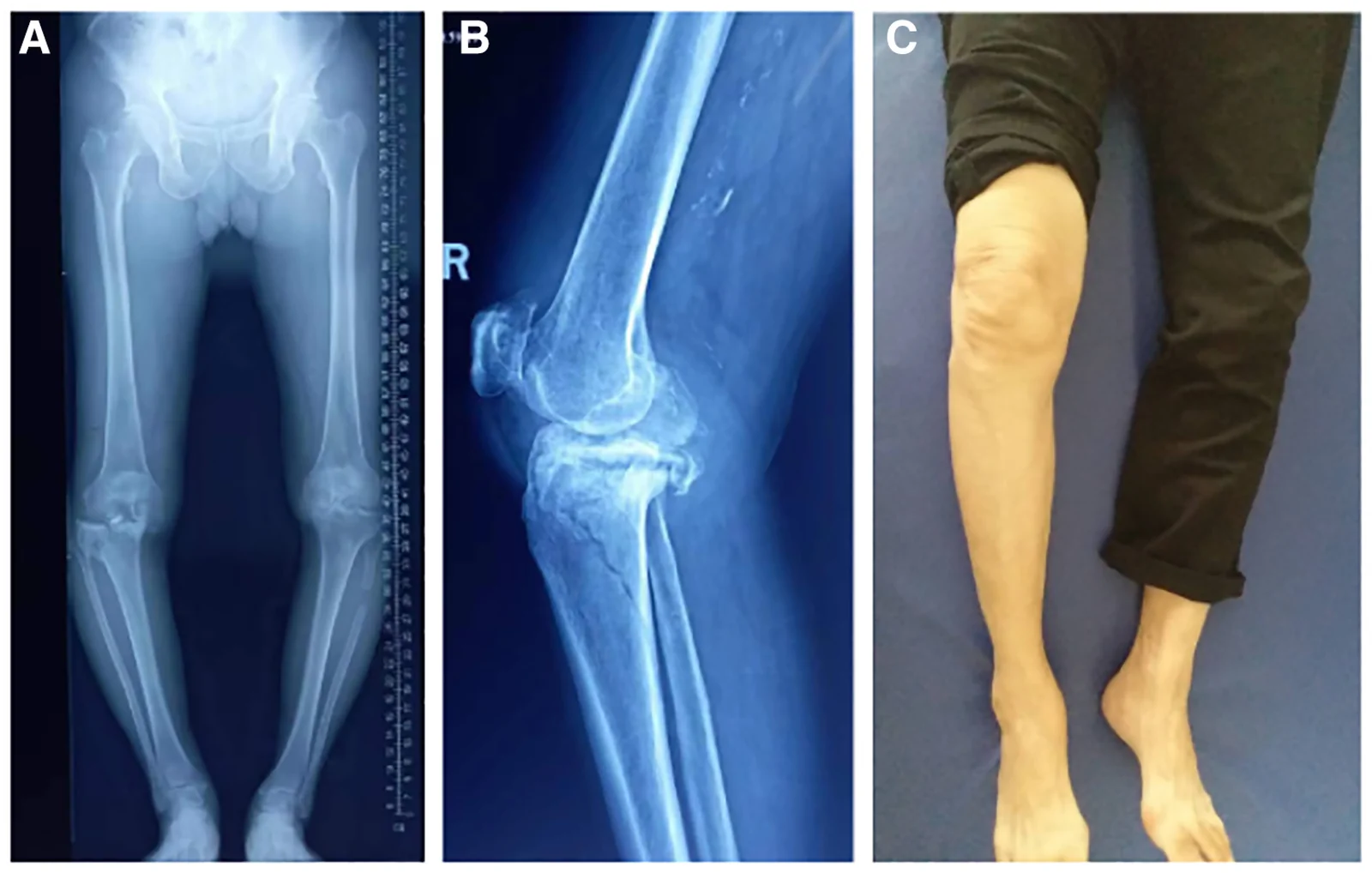
(A) Preoperative full-length radiograph of both lower extremities. (B) Preoperative lateral view radiograph of the right knee. (C) Preoperative anterior view of the patient’s right knee.

The patient had no relevant medical history prior to the injury. There is no history of blood transfusions or allergies to food or medications. and he reports no family history of genetic disorders or infectious diseases. Further physical examination was limited due to limited cooperation. The HSS score was 51 points. Radiographic evaluation demonstrated a tibial plateau fracture nonuion and varus deformity of the right knee joint (Fig. 7).

**Figure 8.**
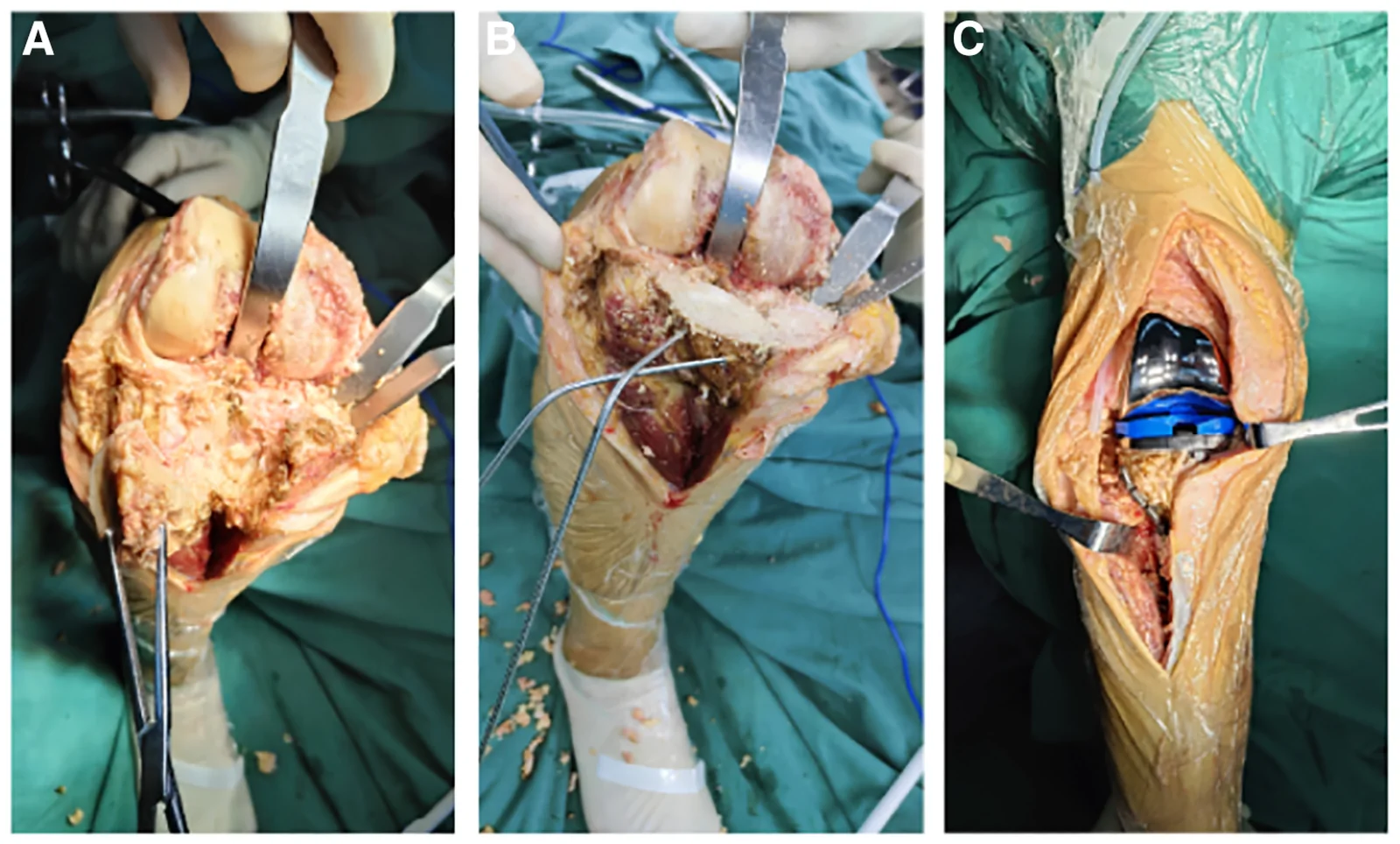
(A) Intraoperative photograph shows the tibial plateau fracture fragment. (B) The fracture fragment is temporarily fixed with Kirschner wires after reduction. (C) The prosthesis and internal fixation devices were implanted successfully.

The diagnoses of this case were nonunited right tibial plateau fracture, post-traumatic osteoarthritis and varus deformity of the right knee joint. Given the lateral tibial plateau fracture, we still employed the LPA as previously described to address this issue. The tibial plateau fracture was first anatomically reduced and provisionally stabilized with 2.0 mm K-wires, followed by measured tibial bone resection using extramedullary alignment guides. The femoral preparation was completed with standard 5° valgus alignment. To enhance tibial component stability, an extension stem was incorporated. After trial prosthesis implantation, satisfactory range of motion and joint stability were confirmed. Prior to definitive component placement, the lateral tibial plateau fracture was stabilized using a lateral tibial plate. Special attention was paid to the lateral positioning of the tibial plate and screw trajectory to avoid interference with prosthesis placement (Fig. 8).

**Figure 9.**
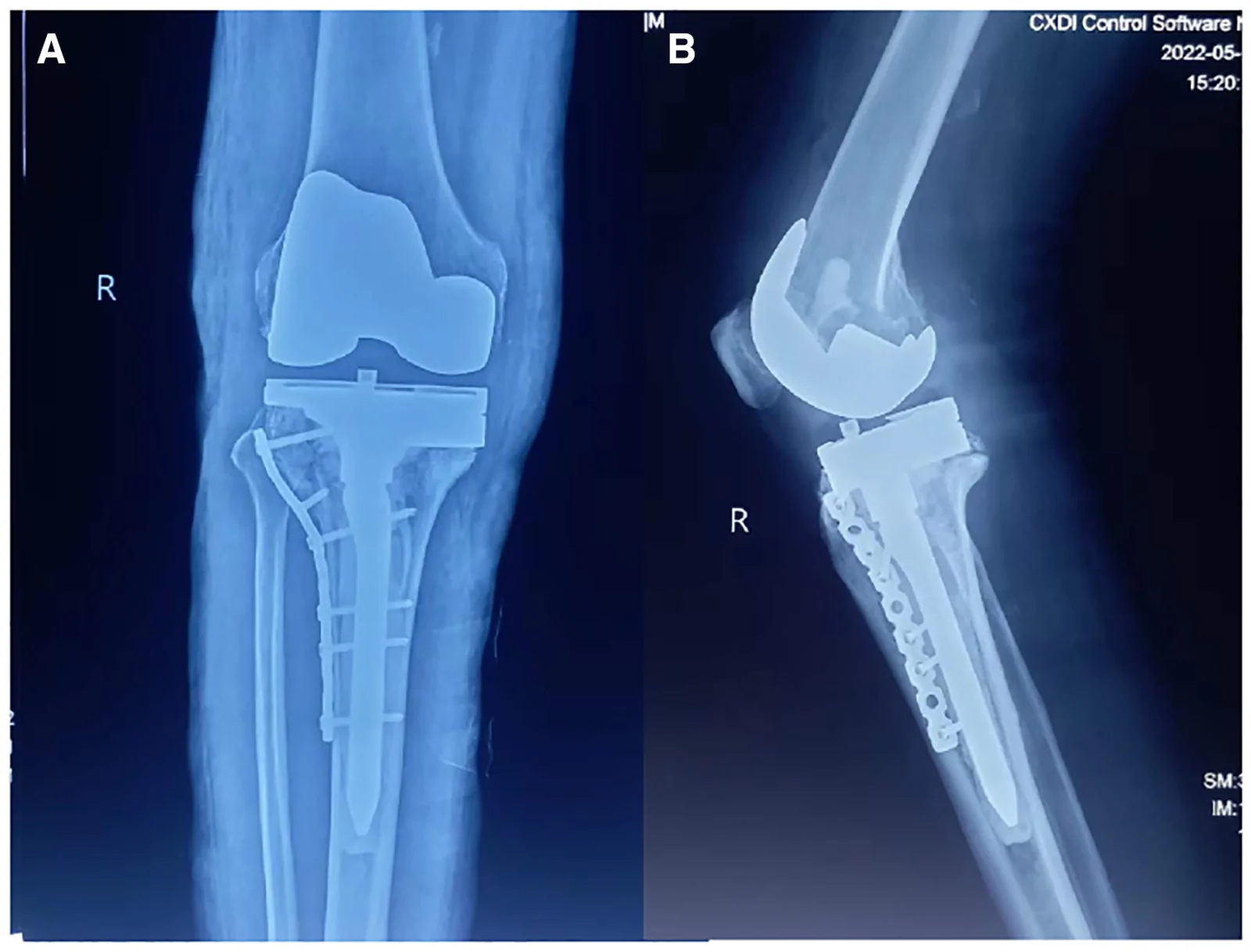
Postoperative anteroposterior and lateral view radiographs of the right knee.

Postoperative radiographic evaluation demonstrated optimal alignment and positioning of the prosthetic components. The patient was followed up for 1-year postoperatively, demonstrating significant improvement in right knee range of motion and marked pain relief. The most recent HSS knee score was 89 points (Fig. 9).

### 2.4. Case 4

A 72-year-old female patient presented with a 10-year history of left knee pain, which had progressively worsened with concomitant functional limitation during the most recent 3 years. The patient had a history of left knee trauma 10 years ago and received a closed lateral tibial osteotomy 3 years ago. She seeks further medical treatment this time.

The patient has a 20-year history of hypertension, regularly takes valsartan orally, and currently has well-controlled blood pressure. There is no history of blood transfusions or allergies to food or medications. Additionally, she reports no family history of genetic disorders or infectious diseases.

**Figure 10.**
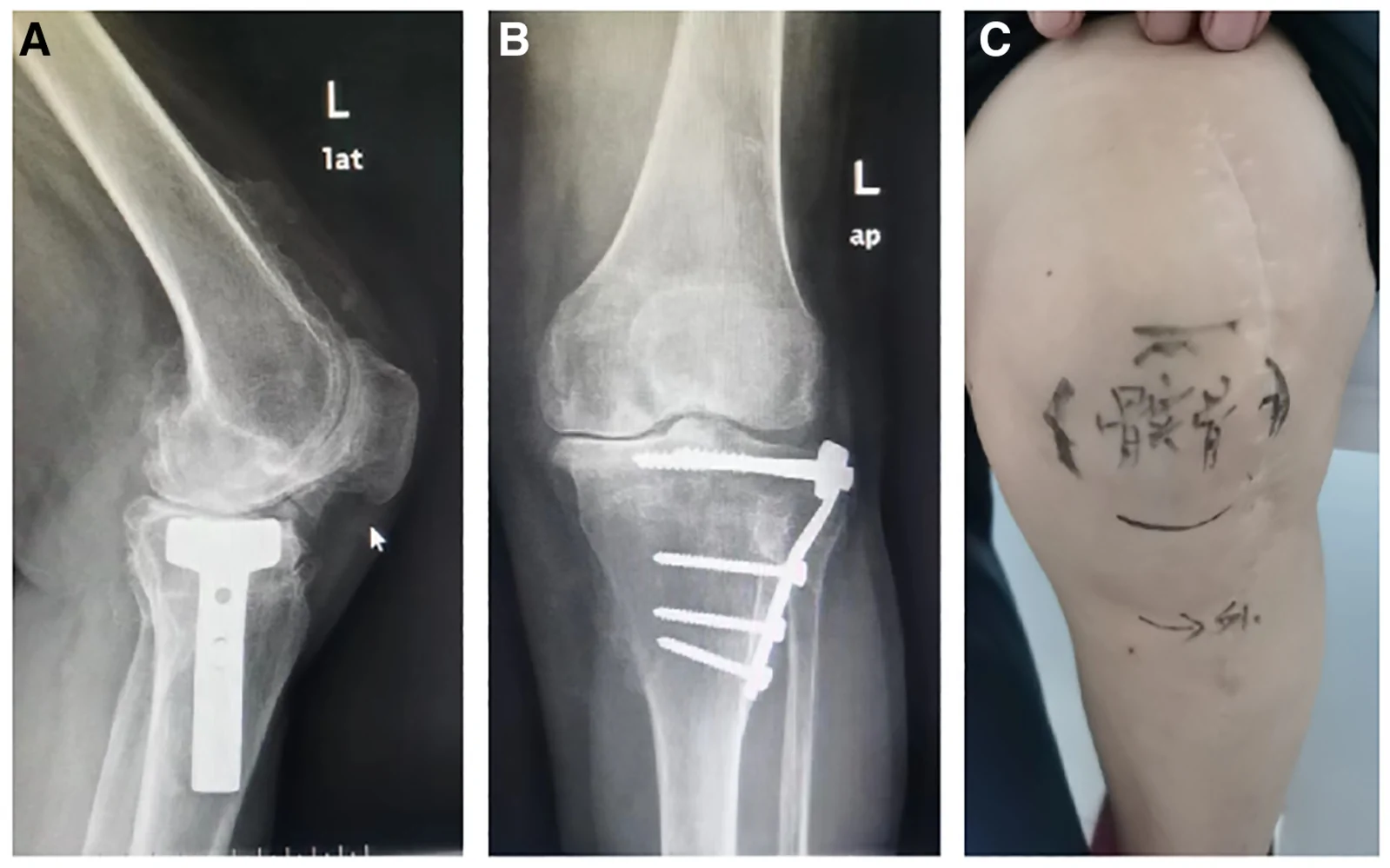
(A) Preoperative anteroposterior view radiograph of the left knee. (B) Preoperative lateral view radiograph of the left knee. (C) Preoperative physical examination revealed a surgical scar on the left lateral knee.

Physical examination revealed a 10 cm longitudinal surgical scar on the lateral aspect of the left knee, with no local erythema, swelling, or sinus tract. Tenderness was noted over both the medial and lateral joint lines, while varus and valgus stress tests were negative. Left knee range of motion: 15 to 45 degrees. The HSS score was 43 points. Radiographic examination reveals post-traumatic arthritis and postoperative changes of the left knee (Fig. 10).

**Figure 11.**
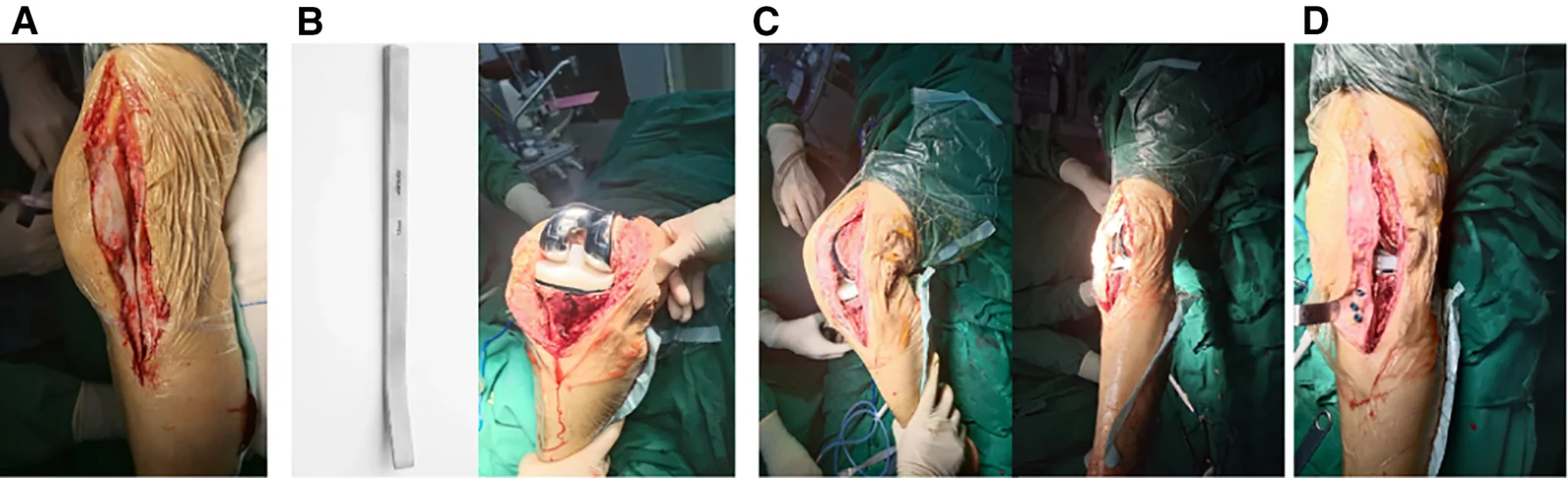
(A) Intraoperative photographs illustrate the exposure achieved through the original incision via LPA. (B) TTO was performed using a curved osteotome. (C) The knee joint demonstrated excellent range of motion after prosthesis placement. (D) The osteotomized fragment was fixed with 3 screws.

The diagnoses of this case were post-traumatic osteoarthritis, stiff knee, and pain after internal fixation surgery of the left knee joint. Given the need to preserve the anterior knee skin’s vascularity while removing the lateral tibial internal fixation device, we still utilized the original incision and accessed the joint cavity via a LPA. Intraoperatively, patellar eversion proved challenging; therefore, a tibial tubercle osteotomy (TTO) was performed to facilitate exposure. Following posterior-stabilized prosthesis implantation, stability and mobility were optimal. The tibial tubercle was secured with cannulated screws, and the incision was closed in layers (Fig. 11).

**Figure 12.**
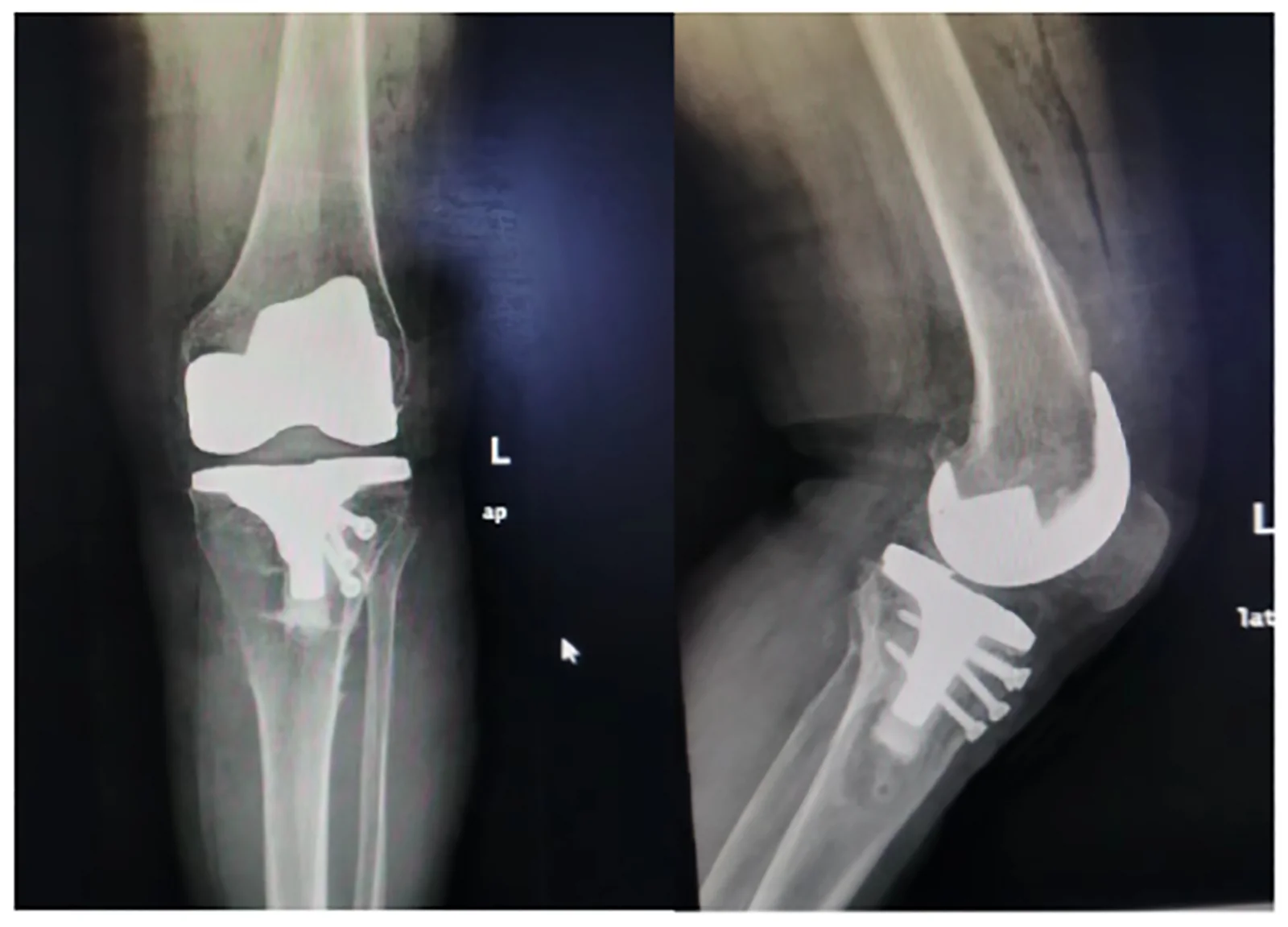
Postoperative anteroposterior and lateral view radiographs of the left knee.

Postoperative radiographic evaluation demonstrated optimal alignment and positioning of the prosthetic components (Fig. 12). The patient was followed up for 1 year postoperatively; the left knee achieved a range of motion of 0° to 90°, with no evidence of proximal migration at the osteotomy site or TTO -related complications. The last HSS knee score was 86 points.

## 3. Discussion

Since Keblish first reported in 1991 that the LPA technique achieved good clinical outcomes for patients undergoing TKA with valgus knees, surgeons have continuously refined this technique. Stehlík utilized coronal Z-plasty of the lateral retinaculum combined with LPA; this method enabled adequate lateral soft tissue release and relieved postoperative complications including patellar ischemia and patellar subluxation, consequently optimizing patellofemoral tracking and lower limb alignment.^[11]^ Satish et al introduced a modified lateral approach for fixed valgus knees, incorporating quadriceps snip, titrated sequential lateral release, and closure with expanded lateral structures. At a mean follow-up of 5 years, radiographic and clinical outcomes demonstrated significant improvement: the average valgus deformity was corrected from a preoperative mean value of 25.4° to 4°, while the KSS score increased from 34 to 95 points.^[12]^

Compared with the traditional MPA, LPA was developed at a later stage and has unique applicable strengths. Published research confirmed that LPA yields favorable effects on valgus deformity correction, including improved surgical efficiency and enhanced postoperative functional outcomes, while maintaining a comparable complication profile.^[8,9]^

Nevertheless, LPA also shows practical value in other special clinical conditions. First, LPA is indicated for most cases with preexisting lateral incisions around the knee joint. Skin blood supply to the anterior knee comes predominantly from the medial side.^[13]^Subsequent adoption of MPA in such cases would disrupt the residual anterior knee arterial anastomotic network, thereby significantly increasing the risk of avascular necrosis of the patella and skin.^[14]^ In Case 1, we presented a patient with a previous lateral knee incision. Although TKA was successfully performed through a medial approach, the patient suffered delayed wound healing that progressed to skin ulcer and infection. We speculate these complications may relate to injury of the arterial anastomotic network. Upon encountering similar cases thereafter, we directly utilized the previous lateral incision, which resulted in favorable wound healing without complications, as shown in Case 4. Secondly, the LPA facilitates the management of concomitant lateral tibial plateau fractures. In Case 3, fracture reduction and plate fixation were achieved through the LPA, which provided excellent surgical accessibility.

Tibial tubercle osteotomy (TTO), as an important surgical technique, plays a crucial role in TKA for stiff knees.^[15,16]^ Adequate exposure is imperative for successful TKA in stiff knees. When the standard LPA proves inadequate for sufficient visualization and surgical maneuverability, adjunctive TTO can be employed to enhance surgical access. Several studies have reported higher rates of osteotomy nonunion and anterior knee pain after TTO; however, none of these complications were observed in our limited case series. Proficient operative technique may serve as the key factor. First, the osteotomy block typically measures approximately 6 cm in length, 2 cm in width, and 1 to 1.5 cm in thickness. The osteotomy is generally performed at a 45°oblique angle, oriented from the proximal-medial to distal-lateral direction. Second, we employ an osteotome for the osteotomy, which effectively avoids the thermal effects associated with oscillating saws while creating a rough osteotomy surface that facilitates reduction and fixation. Maintaining continuity between the osteotomy block and the tibia helps preserve blood supply and enhances postoperative stability. Furthermore, when flipping the bone block, special attention should be paid to protecting the patellar tendon insertion. Finally, after prosthesis implantation, the osteotomy block should be anatomically reduced. Fixation can be achieved using 2 to 3 cortical screws to ensure rigid stabilization and close contact of the osteotomy surfaces. In case 4, we successfully removed the lateral tibial internal fixation, completed the TKA without complications, and achieved satisfactory clinical outcomes through this technique.

Limitations of the present study should be acknowledged. This was a retrospective descriptive case series involving only 4 patients without a control group. Prospective comparative studies with larger cohorts are required to further verify its clinical value.

## 4. Conclusion

The LPA may serve as the preferred surgical alternative for complex knee osteoarthritis under the following clinical scenarios: It is particularly indicated for moderate-to-severe valgus knee deformities; Preferred in cases with preexisting lateral knee surgical scars; Essential when addressing lateral tibial plateau fractures, substantial bone defects, or retained lateral internal fixation devices. requiring management.

## Author contributions

**Conceptualization:** Shuailei Li.

**Data curation:** Boning Yang.

**Software:** Boning Yang.

**Writing – review & editing:** Shuailei Li.

**Writing – original draft:** Boning Yang.
